# Spider hosts (Arachnida, Araneae) and wasp parasitoids (Insecta, Hymenoptera, Ichneumonidae, Ephialtini) matched using DNA barcodes

**DOI:** 10.3897/BDJ.1.e992

**Published:** 2013-09-16

**Authors:** Jeremy A. Miller, J. Dick M. Belgers, Kevin K. Beentjes, Kees Zwakhals, Peter van Helsdingen

**Affiliations:** † Naturalis Biodiversity Center, Leiden, Netherlands; ‡ Wageningen University, Wageningen, Netherlands; § Dr. Dreeslaan 204, Arkel, Netherlands; | European Invertebrate Survey, Leiden, Netherlands

**Keywords:** DNA barcode, host, morphological identification, non-destructive extraction, parasitoid

## Abstract

The study of parasitoids and their hosts suffers from a lack of reliable taxonomic data. We use a combination of morphological characters and DNA sequences to produce taxonomic determinations that can be verified with reference to specimens in an accessible collection and DNA barcode sequences posted to the Barcode of Life database (BOLD). We demonstrate that DNA can be successfully extracted from consumed host spiders and the shed pupal case of a wasp using non-destructive methods. We found *Acrodactyla quadrisculpta* to be a parasitoid of *Tetragnatha montana*; *Zatypota percontatoria* and *Zatypota bohemani* both are parasitoids of *Neottiura bimaculata*. *Zatypota anomala* is a parasitoid of an as yet unidentified host in the family Dictynidae, but the host species may be possible to identify in the future as the library of reference sequences on BOLD continues to grow. The study of parasitoids and their hosts traditionally requires specialized knowledge and techniques, and accumulating data is a slow process. DNA barcoding could allow more professional and amateur naturalists to contribute data to this field of study. A publication venue dedicated to aggregating datasets of all sizes online is well suited to this model of distributed science.

## Introduction

**Figure 1.**
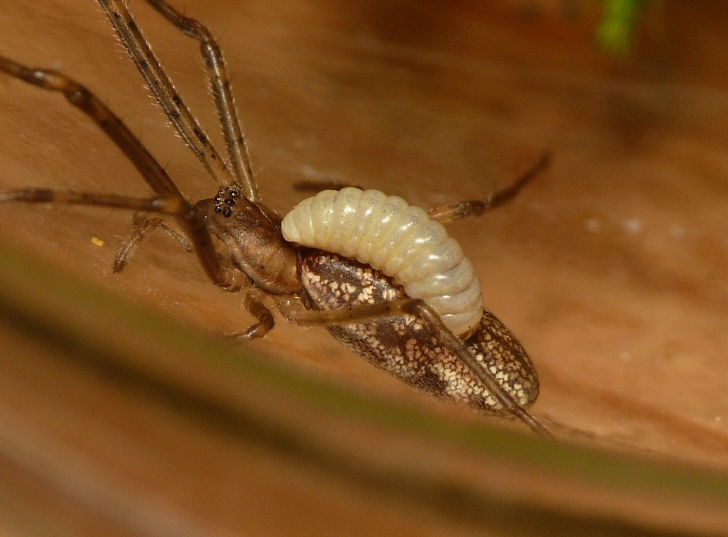
Live *Tetragnatha montana* (RMNH.ARA.14127) parasitized by *Acrodactyla quadrisculpta* larva (RMNH.INS.593867).

Parasitoid wasps are among the most significant enemies of spiders (Foelix 2011). Among the ichneumonid wasps, there are parasitoids of spider eggs, as well as ectoparasitoids of post-embryonic spiders. The "Polysphincta group" of the tribe Ephialtini (also referred to by its junior synonym Polysphinctini) informally refers to those ichneumonids that attack post-embryonic spiders, typically of web-building species. These wasps develop as larvae attached to the abdomen of the spider (Figs 1, 5, 9). This host-parasitoid relationship is distinctive because the mobile spider continues to grow and develop along with the larval parasitoid attached to its abdomen (koinobiont ectoparasitoid). Ultimately, the host is killed and consumed by the larva just prior to pupation. In some cases, the behavior of the spider is modified towards the end of its life to the advantage of the parasitoid (Eberhard 2000).

Most primary data associating spiders and their parasitoid species comes from rearing, i.e., keeping the host spider alive in the lab long enough for the wasp to mature (e.g., Bristow 1941, Nielsen 1923). This approach requires care, hard work, and expertise, and the accumulation of data is a slow process (Fitton et al. 1987). Furthermore, in our experience with "Polysphincta group" wasps, few spider hosts reach maturity before their parasitoids do. This is unfortunate from the perspective of the biologist because most of the best diagnostic morphological characteristics for spider species identification appear only in the adult stage. As a result, positive identification of the host is often elusive and the literature is blemished by dubious host-parasitoid association records (e.g., Shaw 2006).

**Figure 2.**
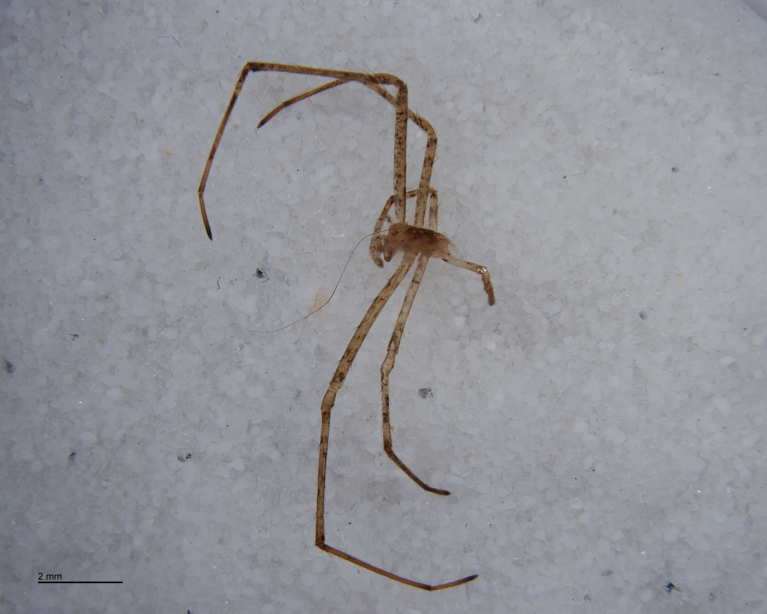
*Tetragnatha montana* (RMNH.ARA.14127) preserved in alcohol after being consumed by *Acrodactyla quadrisculpta* larva (RMNH.INS.593867). Overall view.

**Figure 3.**
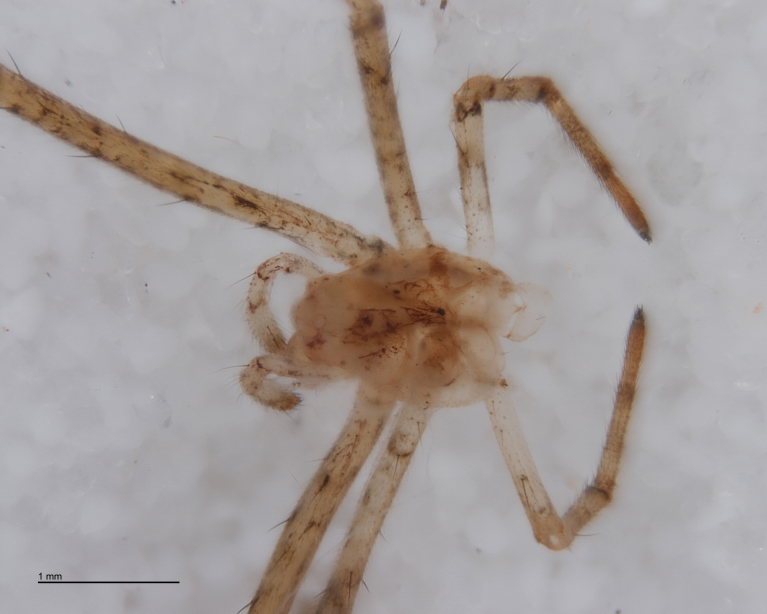
*Tetragnatha montana* (RMNH.ARA.14127) preserved in alcohol after being consumed by *Acrodactyla quadrisculpta* larva (RMNH.INS.593867). Detail of prosoma.

The advent of DNA barcoding offers a path to species determination where traditional morphology falls short. Increasingly, studies of parasitoids and their hosts are turning to DNA-based methods as an alternative or supplement to rearing (Santos et al. 2011, Hrcek et al. 2011, Rougerie et al. 2011, Quicke et al. 2012). Here we apply DNA barcoding techniques to positively associate parasitoids and their hosts. None of the spider hosts in this study reached maturity before being killed by their parasitoid. We successfully obtained DNA barcode sequences from the spider host remains left by the wasp just before pupation (Figs 2, 3, 6, 10, 11, 14, 17). Adult wasps were identified using traditional morphology.

## Materials and methods

In most cases, juvenile spiders observed to be hosting wasp larvae were collected live in the field and taken to the lab for rearing (Figs 1, 5, 9). The adult wasp was allowed to fully develop, killing the host spider in the process. In one record from France, the wasp had already killed its host and pupated inside a loose silk cocoon (Figs 18, 19). In all cases, the host spider died as a subadult and the body was left in poor condition, rendering positive identification by means of traditional morphology unreliable (Figs 2, 3, 6, 10, 11, 14, 17). DNA extractions were performed using the Thermo Labsystems KingFisher extraction robot at the Naturalis Biodiversity Center DNA barcoding facility. For host spiders, DNA was extracted by placing the entire specimen directly (without grinding) in lysis buffer with proteinase K for the three hour incubation step. After incubation, the specimen was returned to ethanol and the extraction continued using the lysis buffer solution. This caused negligible further damage to the specimen. In one case, DNA was also extracted from a wasp larval exuvium by this method (Fig. 7). For adult wasps, one leg was removed as source tissue for DNA extraction. To obtain the standard animal DNA barcode fragment of the mitochondrial cytochrome oxidase I gene (Hebert et al. 2003), PCR was performed using either the primers LCO1490 (5'-GGTCAACAAATCATCATAAAGATATTGG-3') (Folmer et al. 1994) and Chelicerate Reverse 2 (5'-GGATGGCCAAAAAATCAAAATAAATG-3') (Barrett and Hebert 2005) (for spiders and larval exuvium) or a coctail of primers LCO1490 (Hebert et al. 2003) and LepF (5'-ATTCAACCAATCATAAAGATATTGG-3') paired with HCO2198 (5'-TAAACTTCAGGGTGACCAAAAAATCA-3') (Folmer et al. 1994) and LepR1 (5'-TGATTTTTTGGACATCCAGAAGTTTA-3') (Hebert et al. 2003) (for adult wasps). PCR reactions contained 18.75µl mQ, 2.5µ 10x PCR buffer CL, 1.0µl 25mM of each primer, 0.5µl 2.5mM dNTPs and 0.25µl 5U Qiagen Taq. PCR was performed using initial denaturation of 180s at 94°C, followed by 40 cycles of 15s at 94°C, 30s at 50°C and 40s at 72°C, finished with a final extension of 300s at 72°C and pause at 12°C. Sequencing was performed by Macrogen (http://www.macrogen.com). For all barcoded specimens, sequences, images, and collection data were uploaded to the Parasitoid Wasps and Spider Hosts project (PWSH) on the Barcode of Life Database (BOLD; http://www.boldsystems.org/). The "Species Level Barcode Records" search of the BOLD database assisted in host spider identification. Where that was unsuccessful, we resorted to the "All Barcode Records on BOLD" search option and Blast search on NCBI's Genbank (http://www.ncbi.nlm.nih.gov/sites/gquery). Historical literature records of host-parasitoid associations relied on the World Ichneumonoidea database (Yu et al. 2012); primary literature was typically not consulted. For Dutch specimens, latitude and longitude coordinates are converted from the local RD (Rijksdriehoeksmeting) coordinate system; for the French record, coordinates are latitude and longitude. All voucher specimens are deposited in the collection of the Naturalis Biodiversity Center with the exception of one wasp retained in the personal collection of Kees Zwakhals (KZPC).

## Taxon treatments

### Acrodactyla quadrisculpta

(Gravenhorst, 1820)

http://www.boldsystems.org/index.php/TaxBrowser_TaxonPage?subtaxa=hidden&taxid=150402

http://hol.osu.edu/?id=50298

#### Materials

- **Type status:** Other material. **Occurrence:** catalogNumber: RMNH.INS.593867; recordedBy: J. Dick M. Belgers; individualCount: 1; sex: female; associatedOccurrences: RMNH.ARA.14127; associatedSequences: http://www.boldsystems.org/index.php/Public_RecordView?processid=PWSH001-13; **Taxon:** genus: Acrodactyla; specificEpithet: quadrisculpta; scientificNameAuthorship: (Gravenhorst, 1820); **Location:** country: Netherlands; stateProvince: Gelderland; locality: Wageningen, Blauwe Kamer; decimalLatitude: 51.943995; decimalLongitude: 5.61874; coordinateUncertaintyInMeters: 30; **Event:** samplingProtocol: found (by beating) on fijnspar (Norway Spruce, Picea abies); eventDate: 04/17/2012; **Record Level:** institutionCode: RMNH; basisOfRecord: specimen
- **Type status:** Other material. **Occurrence:** catalogNumber: RMNH.INS.593868; recordedBy: J. Dick M. Belgers; individualCount: 1; sex: male; associatedOccurrences: RMNH.ARA.14128; associatedSequences: http://www.boldsystems.org/index.php/Public_RecordView?processid=PWSH002-13; **Taxon:** genus: Acrodactyla; specificEpithet: quadrisculpta; scientificNameAuthorship: (Gravenhorst, 1820); **Location:** country: Netherlands; stateProvince: Gelderland; locality: Wageningen, Blauwe Kamer; decimalLatitude: 51.943995; decimalLongitude: 5.61874; coordinateUncertaintyInMeters: 30; **Event:** samplingProtocol: found (by beating) on fijnspar (Norway Spruce, Picea abies); eventDate: 04/19/2012; **Record Level:** institutionCode: RMNH; basisOfRecord: specimen
- **Type status:** Other material. **Occurrence:** recordedBy: J. Dick M. Belgers; individualCount: 1; sex: male; associatedOccurrences: RMNH.ARA.14129; **Taxon:** genus: Acrodactyla; specificEpithet: quadrisculpta; scientificNameAuthorship: (Gravenhorst, 1820); **Location:** country: Netherlands; stateProvince: Gelderland; locality: Wageningen, Blauwe Kamer; decimalLatitude: 51.943995; decimalLongitude: 5.61874; coordinateUncertaintyInMeters: 30; **Event:** samplingProtocol: found (by beating) on fijnspar (Norway Spruce, Picea abies); eventDate: 04/19/2012; **Record Level:** institutionCode: KZPC; basisOfRecord: specimen

#### Notes

Shaw (2006) notes the possibility of some confusion in the literature concerning this parasitoid and the morphologically similar *Acrodactyla carinator* (Aubert, 1965). Errors in identification of the parasitoid and/or host may be obscuring the true host specificity of these wasps in some parts of their distribution.

**Figure 4.**
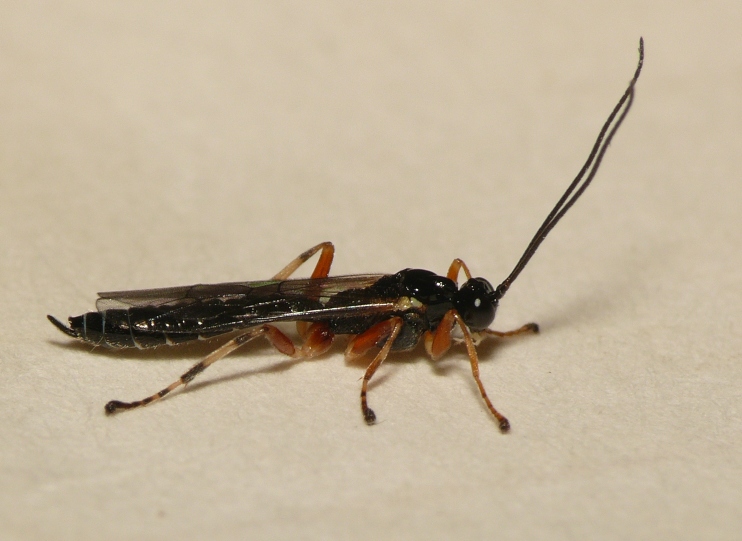
Live adult female *Acrodactyla quadrisculpta* (RMNH.INS.593867).

**Figure 5.**
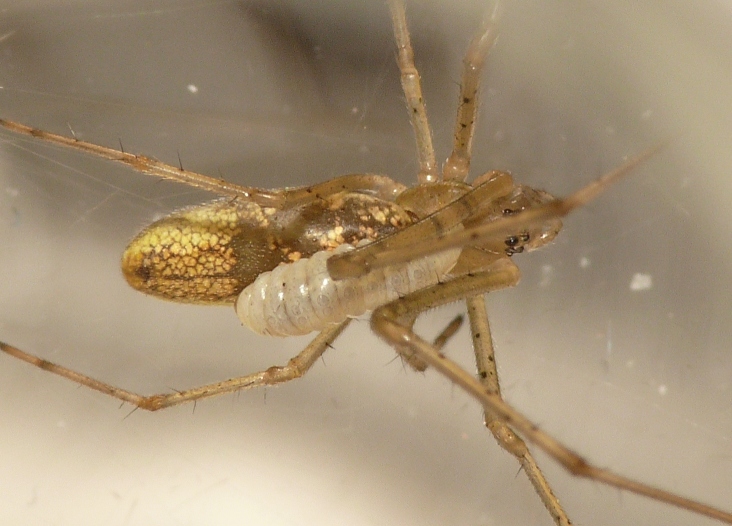
Live *Tetragnatha montana* (RMNH.ARA.14128) parasitized by *Acrodactyla quadrisculpta* larva (RMNH.INS.593868).

**Figure 6.**
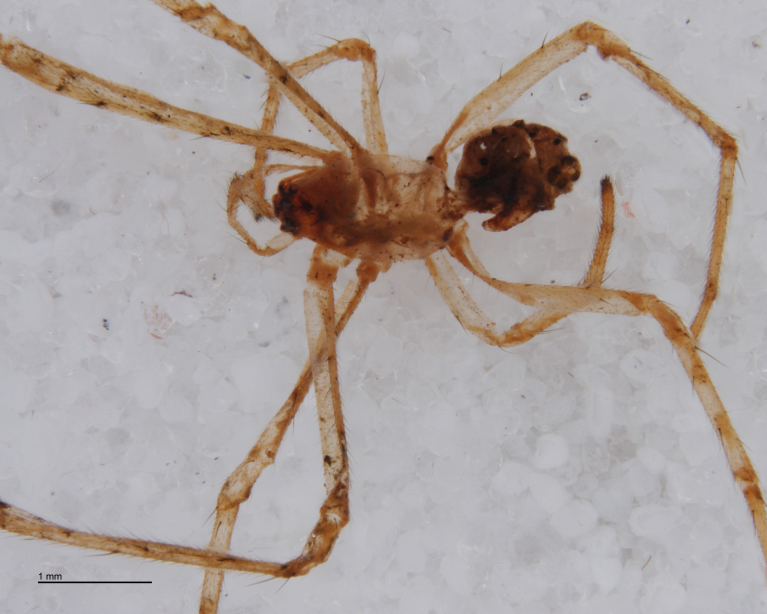
*Tetragnatha montana* (RMNH.ARA.14128) preserved in alcohol after being consumed by *Acrodactyla quadrisculpta* larva (RMNH.INS.593868).

**Figure 7.**
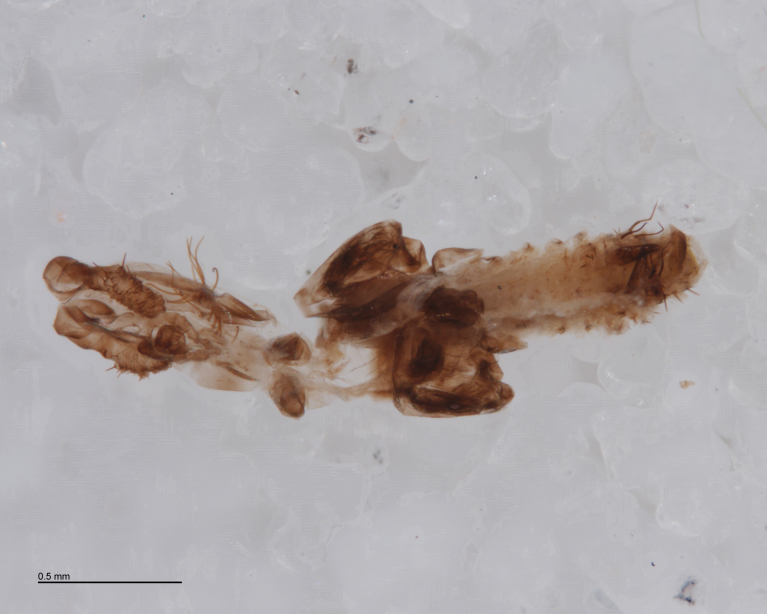
Larval exuvium of *Acrodactyla quadrisculpta* (RMNH.INS.593868). DNA barcode sequence was obtained from this specimen.

**Figure 8.**
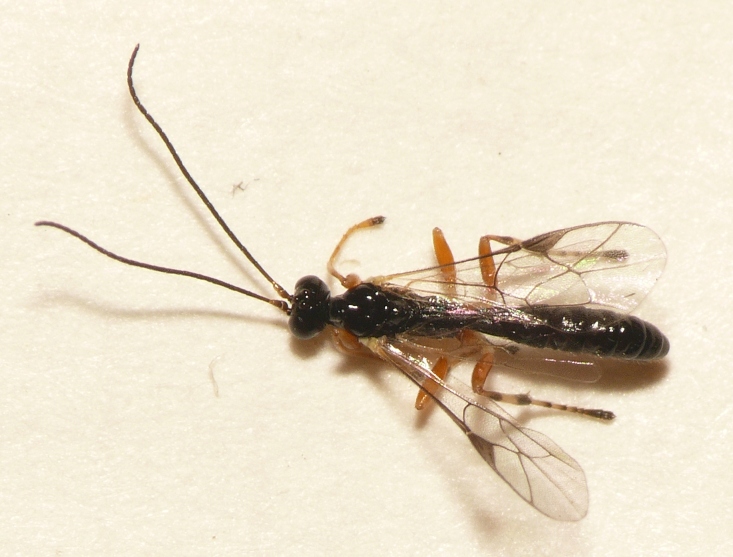
Live adult male *Acrodactyla quadrisculpta* (RMNH.INS.593868).

**Figure 9.**
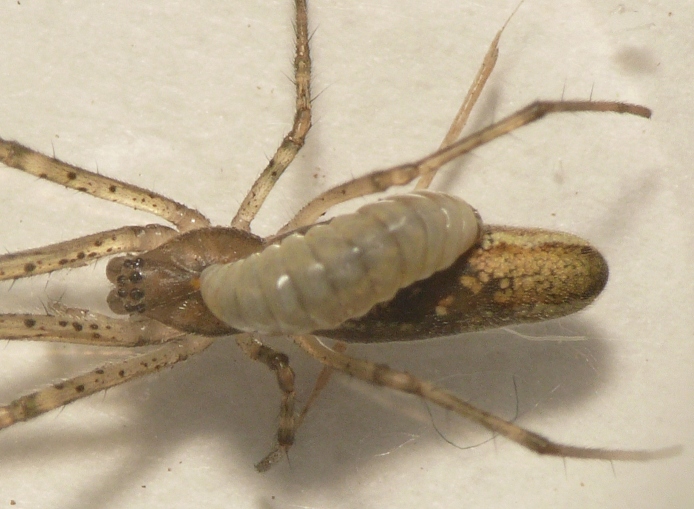
Live *Tetragnatha montana* (RMNH.ARA.14129) parasitized by *Acrodactyla quadrisculpta* larva (KZPC).

**Figure 10.**
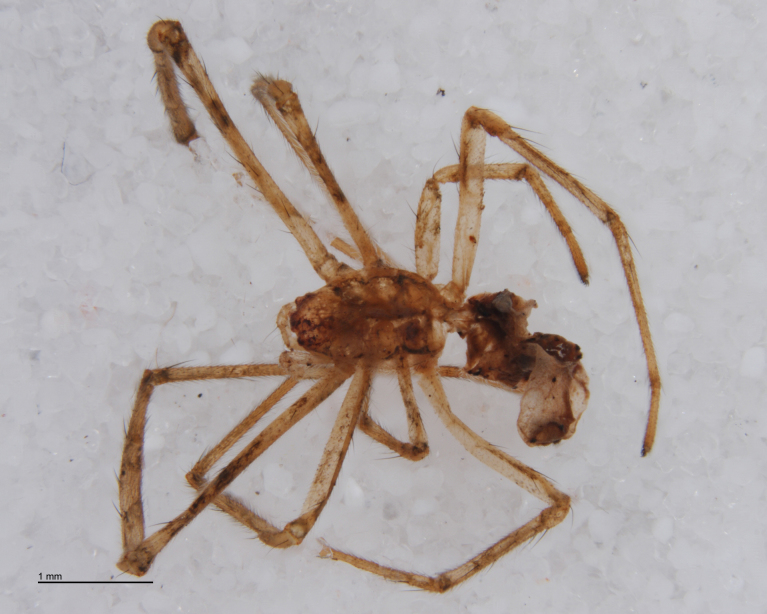
*Tetragnatha montana* (RMNH.ARA.14129) preserved in alcohol after being consumed by *Acrodactyla quadrisculpta* larva.

Adult wasps were identified as *Acrodactyla quadrisculpta* (Figs 4, 8) by an experienced ichneumonid taxonomist based on morphological characteristics with reference to the taxonomic literature. DNA barcodes derived from two adult specimens (RMNH.INS.593867 and RMNH.INS.593868) plus the larval exuvium (Fig. 7) were used to query the BOLD database; no match was found. The barcode sequence from the larval exuvium was identical to that of the adult (RMNH.INS.593868) except that the last 9 bases on the 3' end were not sequenced. A subsequent Blast search of NCBI's GenBank found sequences identified as *Acrodactyla quadrisculptata* among the closest matches. However, the closest matching sequences scored only 93% similarity, lower than expected for most conspecific DNA barcodes. Of the 12 sequences with this similarity score, three were identified as *Acrodactyla quadrisculpta*; the remaining sequences were less precisely identified but the taxonomic information was not in conflict with *Acrodactyla quadrisculpta*. Most if not all of the closest matching DNA barcode sequences were derived from specimens collected in Manitoba, Canada. The resolution of these facts might lie in a high genetic diversity within this widespread species, or taxonomic error at some level. One specimen of *Acrodactyla quadrisculpta* was not sequenced and remains in the personal collection of Kees Zwakhals (KZPC).

### Tetragnatha montana

Simon, 1874

http://www.boldsystems.org/index.php/TaxBrowser_TaxonPage?subtaxa=hidden&taxid=178345

http://www.araneae.unibe.ch/data/846/Tetragnatha_montana

#### Materials

- **Type status:** Other material. **Occurrence:** catalogNumber: RMNH.ARA.14127; recordedBy: J. Dick M. Belgers; individualCount: 1; sex: female; lifeStage: juvenile; associatedOccurrences: RMNH.INS.593867; associatedSequences: http://www.boldsystems.org/index.php/Public_RecordView?processid=NLARA239-12; **Taxon:** genus: Tetragnatha; specificEpithet: montana; scientificNameAuthorship: Simon, 1874; **Location:** country: Netherlands; stateProvince: Gelderland; locality: Wageningen, Blauwe Kamer; decimalLatitude: 51.943995; decimalLongitude: 5.61874; coordinateUncertaintyInMeters: 30; **Event:** samplingProtocol: found (by beating) on fijnspar (Norway Spruce, Picea abies); eventDate: 03/30/2012; **Record Level:** institutionCode: RMNH; basisOfRecord: specimen
- **Type status:** Other material. **Occurrence:** catalogNumber: RMNH.ARA.14128; recordedBy: J. Dick M. Belgers; individualCount: 1; sex: female; lifeStage: juvenile; associatedOccurrences: RMNH.INS.593868; associatedSequences: http://www.boldsystems.org/index.php/Public_RecordView?processid=NLARA250-12; **Taxon:** genus: Tetragnatha; specificEpithet: montana; scientificNameAuthorship: Simon, 1874; **Location:** country: Netherlands; stateProvince: Gelderland; locality: Wageningen, Blauwe Kamer; decimalLatitude: 51.943995; decimalLongitude: 5.61874; coordinateUncertaintyInMeters: 30; **Event:** samplingProtocol: found (by beating) on fijnspar (Norway Spruce, Picea abies); eventDate: 04/01/2012; **Record Level:** institutionCode: RMNH; basisOfRecord: specimen
- **Type status:** Other material. **Occurrence:** catalogNumber: RMNH.ARA.14129; recordedBy: J. Dick M. Belgers; individualCount: 1; sex: female; lifeStage: juvenile; associatedSequences: http://www.boldsystems.org/index.php/Public_RecordView?processid=NLARA260-12; **Taxon:** genus: Tetragnatha; specificEpithet: montana; scientificNameAuthorship: Simon, 1874; **Location:** country: Netherlands; stateProvince: Gelderland; locality: Wageningen, Blauwe Kamer; decimalLatitude: 51.943995; decimalLongitude: 5.61874; coordinateUncertaintyInMeters: 30; **Event:** samplingProtocol: found (by beating) on fijnspar (Norway Spruce, Picea abies); eventDate: 04/01/2012; **Record Level:** institutionCode: RMNH; basisOfRecord: specimen

#### Notes

A search of the BOLD database indicated that the host for all three *Acrodactyla quadrisculpta* specimens was *Tetragnatha montana* Simon, 1874. Host sequences scored 98.3%-100% similarity with 21 other data points identified as *Tetragnatha montana* with DNA barcode sequences available in BOLD (all private or early-release at the time of writing). Some of the 100% matches were *Tetragnatha montana* specimens sequenced as part of a DNA barcoding study on Dutch spiders (Miller et al. 2013). *Acrodactyla quadrisculpta* has been associated historically with a number of host species in the genus *Tetragnatha* including *Tetragnatha montana* (Yu et al. 2012).

### Zatypota percontatoria

(Müller, 1776)

http://www.boldsystems.org/index.php/TaxBrowser_TaxonPage?subtaxa=hidden&taxid=449209

http://hol.osu.edu/index.html?id=50450

#### Materials

- **Type status:** Other material. **Occurrence:** catalogNumber: RMNH.INS.593327; recordedBy: J. Dick M. Belgers; individualCount: 1; sex: female; lifeStage: adult; associatedOccurrences: RMNH.ARA.14036; associatedSequences: http://www.boldsystems.org/index.php/Public_RecordView?processid=PWSH004-13; **Location:** country: Netherlands; stateProvince: Gelderland; locality: Wageningen, Blauwe Kamer; decimalLatitude: 51.9428; decimalLongitude: 5.631533; coordinateUncertaintyInMeters: 30; **Event:** eventDate: 2012-09-01; **Record Level:** institutionCode: RMNH; basisOfRecord: specimen

### Zatypota bohemani

(Holmgren, 1860)

http://www.boldsystems.org/index.php/TaxBrowser_TaxonPage?subtaxa=hidden&taxid=469733

http://hol.osu.edu/index.html?id=50427

#### Materials

- **Type status:** Other material. **Occurrence:** catalogNumber: RMNH.INS.593328; recordedBy: J. Dick M. Belgers; individualCount: 1; sex: female; lifeStage: adult; associatedOccurrences: RMNH.ARA.14037; associatedSequences: http://www.boldsystems.org/index.php/Public_RecordView?processid=PWSH006-13; **Location:** country: Netherlands; stateProvince: Gelderland; locality: Wageningen, Blauwe Kamer; decimalLatitude: 51.94372; decimalLongitude: 5.619903; coordinateUncertaintyInMeters: 30; **Event:** eventDate: 2012-07-05; **Record Level:** institutionCode: RMNH; basisOfRecord: specimen

### Neottiura bimaculata

(Linnaeus, 1767)

http://www.boldsystems.org/index.php/TaxBrowser_TaxonPage?subtaxa=hidden&taxid=29775

http://www.araneae.unibe.ch/data/56/Neottiura_bimaculata

#### Materials

- **Type status:** Other material. **Occurrence:** catalogNumber: RMNH.ARA.14036; recordedBy: J. Dick M. Belgers; individualCount: 1; sex: female; lifeStage: juvenile; associatedOccurrences: RMNH.INS.593327; associatedSequences: http://www.boldsystems.org/index.php/Public_RecordView?processid=PWSH005-13; **Location:** country: Netherlands; stateProvince: Gelderland; locality: Wageningen, Blauwe Kamer; decimalLatitude: 51.9428; decimalLongitude: 5.631533; coordinateUncertaintyInMeters: 30; **Event:** eventDate: 2012-08-12; **Record Level:** institutionCode: RMNH; basisOfRecord: specimen
- **Type status:** Other material. **Occurrence:** catalogNumber: RMNH.ARA.14037; recordedBy: J. Dick M. Belgers; individualCount: 1; sex: female; lifeStage: juvenile; associatedOccurrences: RMNH.INS.593328; associatedSequences: http://www.boldsystems.org/index.php/Public_RecordView?processid=PWSH007-13; **Location:** country: Netherlands; stateProvince: Gelderland; locality: Wageningen, Blauwe Kamer; decimalLatitude: 51.94372; decimalLongitude: 5.619903; coordinateUncertaintyInMeters: 30; **Event:** eventDate: 2012-06-14; **Record Level:** institutionCode: RMNH; basisOfRecord: specimen

#### Notes

**Figure 11.**
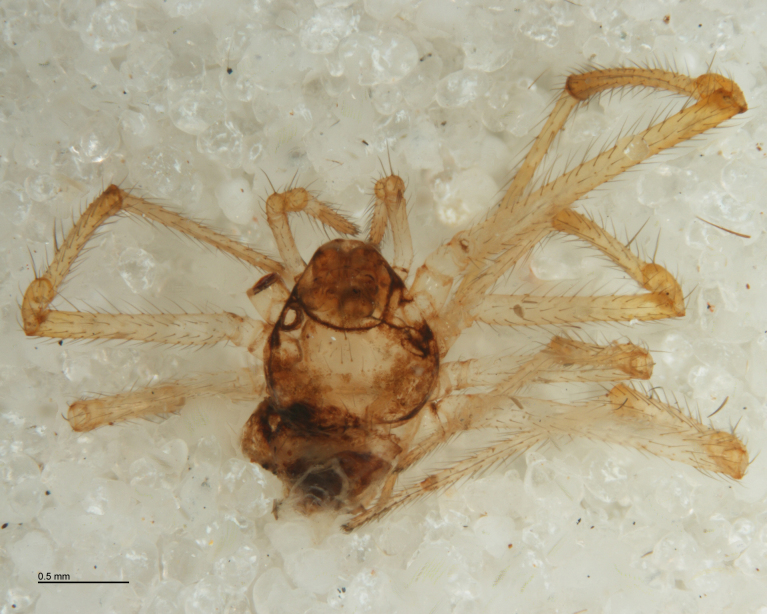
*Neottiura bimaculata* (RMNH.ARA.14036) preserved in alcohol after being consumed by *Zatypota percontatoria* larva (RMNH.INS.593327).

**Figure 12.**
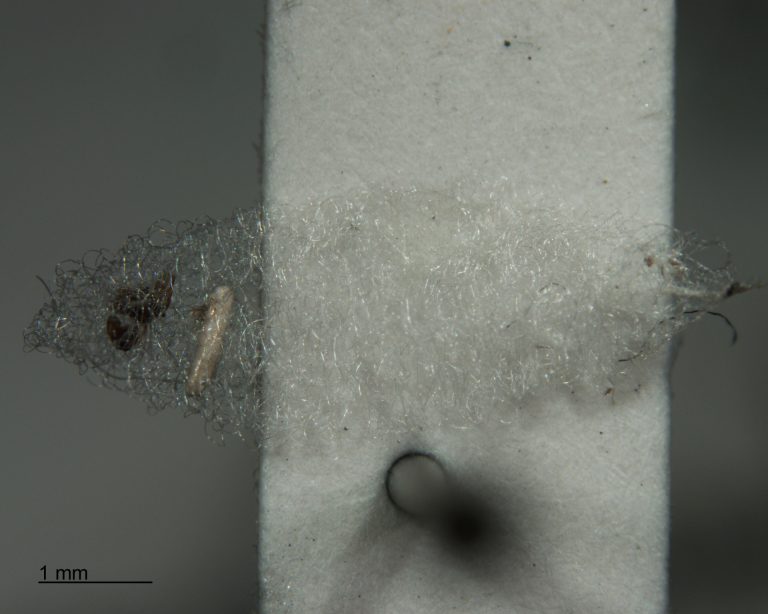
Silk coccoon of *Zatypota percontatoria* (RMNH.INS.593327), preserved specimen.

**Figure 13.**
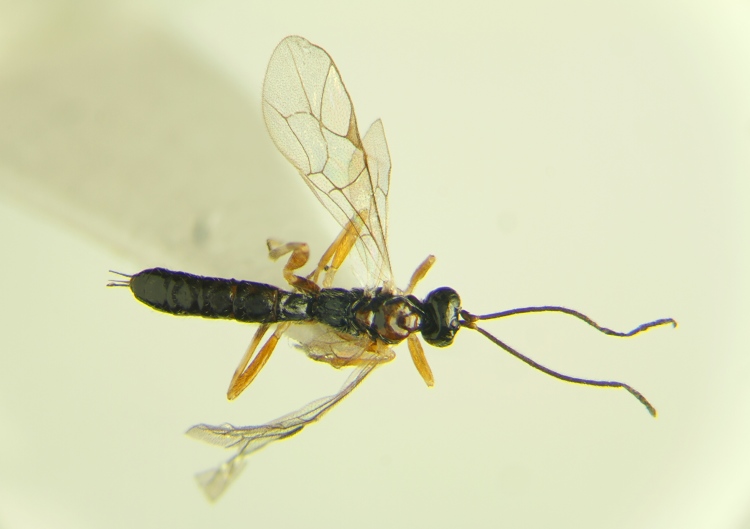
Adult *Zatypota percontatoria* (RMNH.INS.593327), specimen.

**Figure 14.**
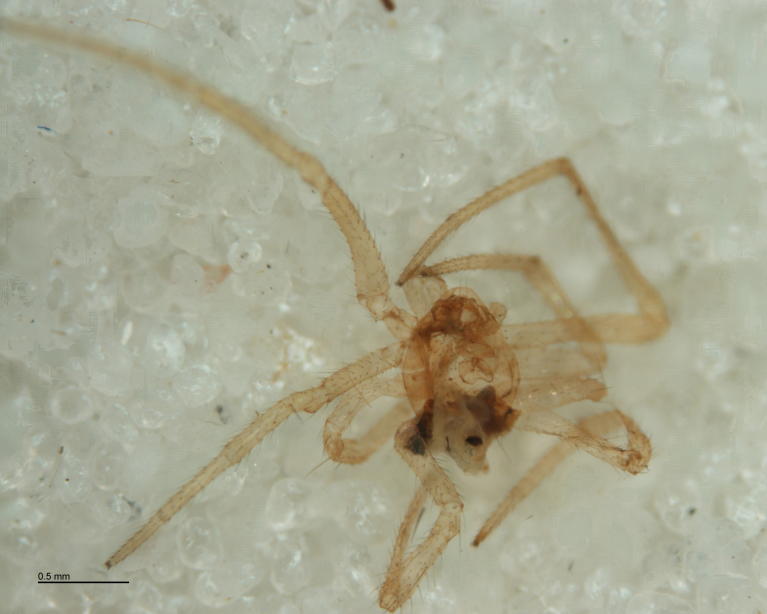
*Neottiura bimaculata* (RMNH.ARA.14037) preserved in alcohol after being consumed by *Zatypota bohemani* larva (RMNH.INS.593328).

**Figure 15.**
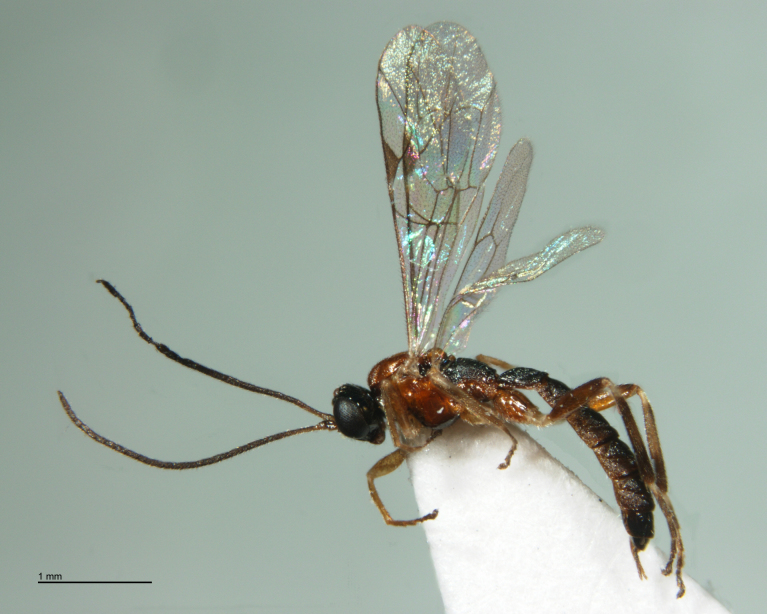
Adult female *Zatypota bohemani* (RMNH.INS.593328), specimen.

**Figure 16.**
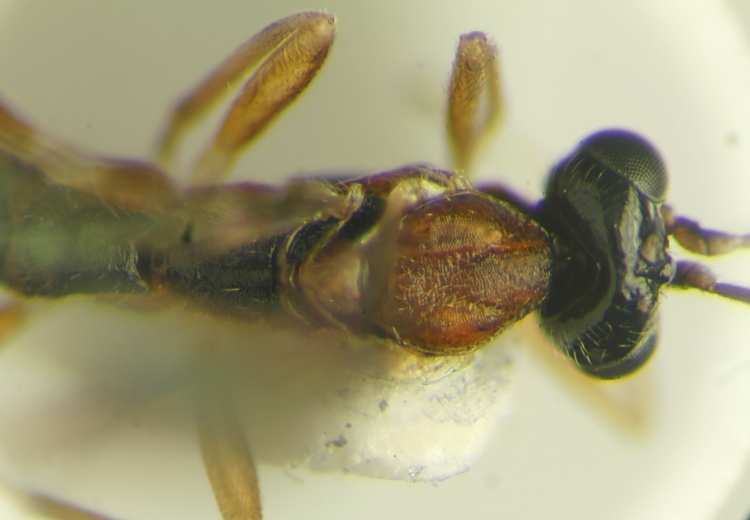
Adult female *Zatypota bohemani* (RMNH.INS.593328), specimen, detail of pronotum.

A search of the BOLD database indicated that these two hosts are *Neottiura bimaculata* (Linnaeus, 1767) (Figs 11, 14). Host sequences scored 96.8%-99.5% similarity with 20 other data points identified as *Neottiura bimaculata* with DNA barcode sequences available in BOLD including three specimens from a study on Dutch spiders (Miller et al. 2013). Two parasitoid species were found to be associated with *Neottiura bimaculata*: *Zatypota percontatoria* (Figs 12, 13) and *Zatypota bohemani* (Figs 15, 16). Neither parasitoid had been associated previously with *Neottiura bimaculata* hosts (Yu et al. 2012).

### Zatypota anomala

(Gravenhorst, 1820)

http://www.boldsystems.org/index.php/Taxbrowser_Taxonpage?taxid=482017

http://www.boldsystems.org/index.php/Taxbrowser_Taxonpage?taxid=449222

http://hol.osu.edu/index.html?id=50410

Zatypota anomala This species was transfered from the genus *Sinarachna* to *Zatypota* by Zwakhals (2006), but this act has not been consistently reflected by some online resources. Hymenoptera Online lists this species under *Sinarachna*. BOLD has data under both *Sinarachna anomala* and *Zatypota anomala*.

#### Materials

- **Type status:** Other material. **Occurrence:** catalogNumber: RMNH.INS.593866; recordedBy: Hélène Dumas; individualCount: 1; sex: female; lifeStage: adult; associatedOccurrences: RMNH.ARA.14254; **Location:** country: France; stateProvince: Bouches-du-Rhône; verbatimLocality: La Ciotat; decimalLatitude: 43.198642; decimalLongitude: 5.631474; coordinateUncertaintyInMeters: 30; **Event:** samplingProtocol: in my semi-wild garden under a leaf of Pittosporum tobira, at about 1.3 m high, cocoon with wasp pupa and dead host found 13 July 2012, cut leaf with cocoon kept outside, sheltered from sun and rain; eventDate: 2012-07-20; **Record Level:** institutionCode: RMNH; basisOfRecord: specimen

### Dictynidae sp.

http://www.boldsystems.org/index.php/TaxBrowser_TaxonPage?subtaxa=hidden&taxid=1343

http://www.araneae.unibe.ch/list/gen/taxId/17/Dictynidae

#### Materials

- **Type status:** Other material. **Occurrence:** catalogNumber: RMNH.ARA.14254; recordedBy: Hélène Dumas; individualCount: 1; sex: female; lifeStage: juvenile; associatedOccurrences: RMNH.INS.593866; associatedSequences: http://www.boldsystems.org/index.php/Public_RecordView?processid=PWSH009-13; **Location:** country: France; stateProvince: Bouches-du-Rhône; verbatimLocality: La Ciotat; decimalLatitude: 43.198642; decimalLongitude: 5.631474; coordinateUncertaintyInMeters: 30; **Event:** samplingProtocol: in my semi-wild garden under a leaf of Pittosporum tobira, at about 1.3 m high, cocoon with wasp pupa and dead host found 13 July 2012, cut leaf with cocoon kept outside, sheltered from sun and rain; eventDate: 2012-07-13; **Record Level:** institutionCode: RMNH; basisOfRecord: specimen

#### Notes

**Figure 17.**
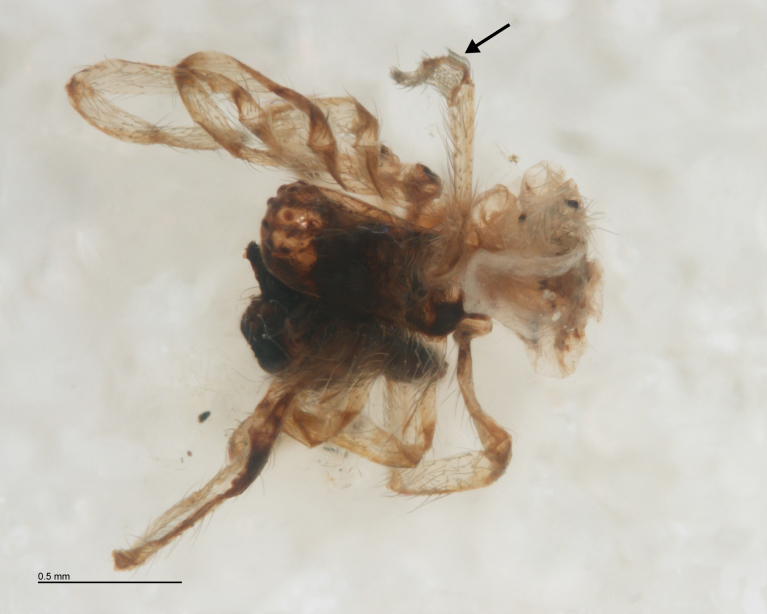
Dictynidae sp. (RMNH.ARA.14254) preserved in alcohol after being consumed by *Zatypota anomala* larva (RMNH.INS.593866). Arrow indicates calamistrum, a morphological structure found in some dictynid species, but relatively few other European spiders.

**Figure 18.**
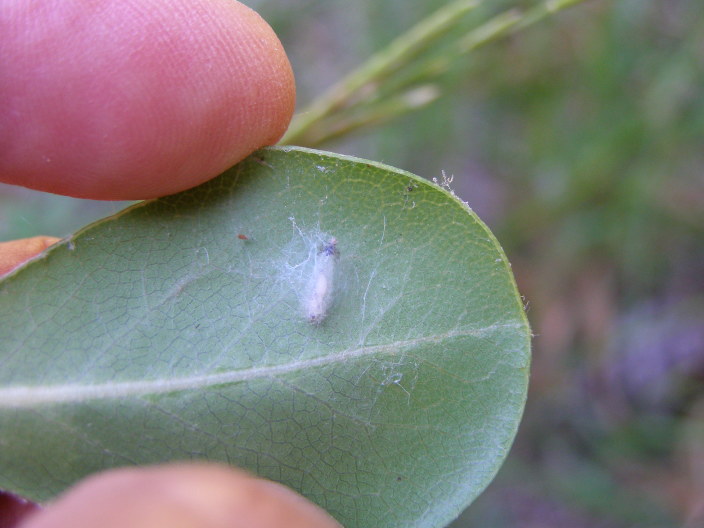
*Zatypota anomala* (RMNH.INS.593866) undergoing metamorphosis in the field.

**Figure 19.**
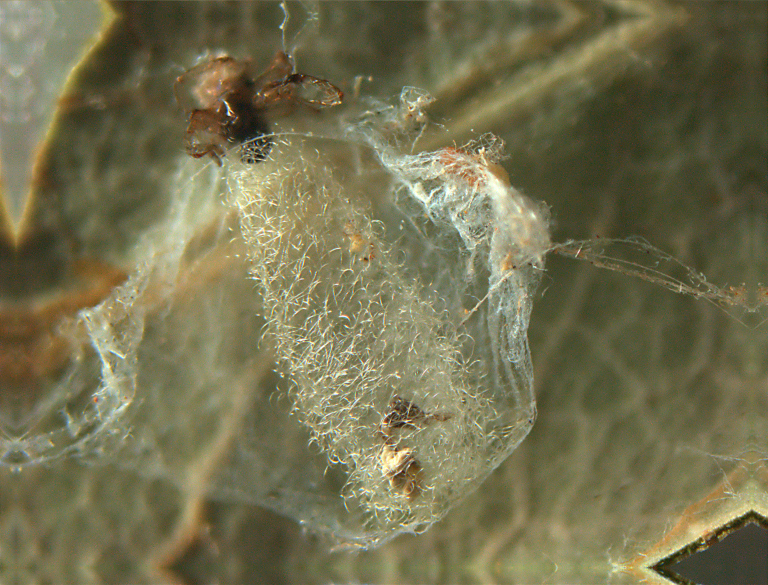
Silk coccoon of *Zatypota anomala* (RMNH.INS.593866) in the field.

**Figure 20.**
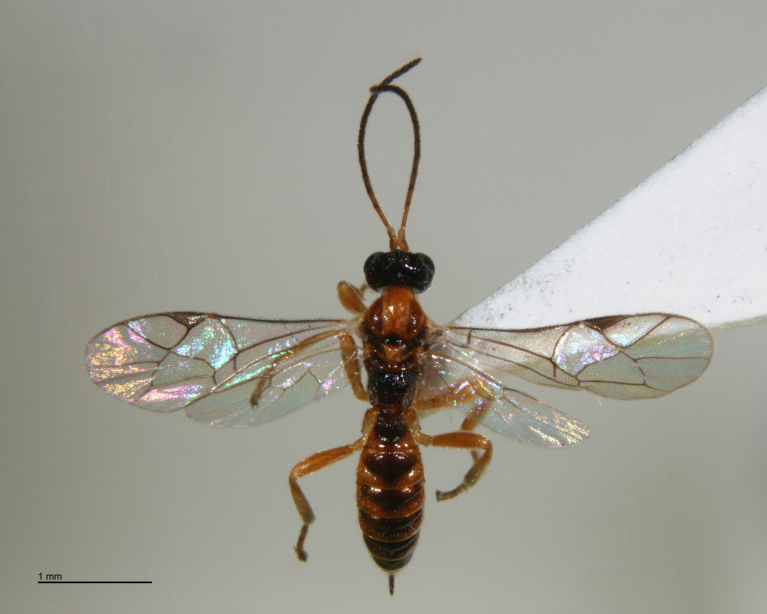
Adult female *Zatypota anomala* (RMNH.INS.593866), specimen.

The host specimen could not be precisely identified using either morphology or the Species Level Barcode Record search of the sequence library available on BOLD at the time of this writing. A more general search of BOLD using the All Barcode Records search option returned a closest match (95.26% similarity) with the dictynid *Nigma walckenaeri*. A query of Genbank returned a closest match (88%) with *Dictyna latens*. A calamistrum is visible on the fourth metatarsus of the presereved host specimen (Fig. 17). The calamistrum is an organ involved in the spinning of cribellate silk (Köhler and Vollrath 1995, Opell 1998). The presence of a cribellum alone eliminates the vast majority of European spider taxa. In combination with the overall size and shape, we conclude this host belongs to the spider family Dictynidae. The World Ichneumonoidea database on Taxapad indicates that *Zatypota anomala* (Fig. 20) has been associated with dictynid spider hosts (Yu et al. 2012). The incompletely identified host DNA barcode sequence has been deposited in BOLD. As the library of reference sequences grows, it may become possible to identify this host to species. An attempt to barcode the parasitoid *Zatypota anomala* was not successful.

## Discussion

DNA barcoding is best thought of as a supplement, not a replacement, for traditional methods of taxonomic identification because both approaches have different, often complementary strengths and limitations (Dayrat 2005, Will et al. 2005). There are now many examples where the integration of DNA sequence data and morphological data have advanced knowledge in ways that would not have been possible without this synergy (Riedel et al. 2013, Paquin and Hedin 2004). The study presented here is a case where morphology alone is not adequate because of the physical condition and developmental stage of the host at the time of death. Fortunately, we found that the condition of the host specimens did not prevent the generation of DNA barcode sequence data.

Major advances in the study of host-parasitoid relationships require primary data from a wide taxonomic and geographic range. Questions about host specificity and changes in host-parasitoid relationships across large spatial scales are two important topics hamstrung by the scarcity of primary data. But the slow pace of traditional approaches means that few specialists can be dedicated to this area of study. These days, DNA barcoding requires far less expertise, opening the door for more non-specialists to contribute data in small quantities. Depositing these data online in community databases like BOLD, and publishing results in an internet savvy journal dedicated to aggregating datasets of all sizes, offers a strategy for advancing knowledge of host-parasitoid relationships not available to previous generations of scientists.

## Supplementary Material

XML Treatment for Acrodactyla quadrisculpta

XML Treatment for Tetragnatha montana

XML Treatment for Zatypota percontatoria

XML Treatment for Zatypota bohemani

XML Treatment for Neottiura bimaculata

XML Treatment for Zatypota anomala

XML Treatment for Dictynidae sp.
